# Isolation, identification, and genomic analysis of *Fusarium verticillioides* CMGF-RB13 causing strawberry root rot

**DOI:** 10.3389/fmicb.2026.1958307

**Published:** 2026-09-30

**Authors:** Haohao Yan, Chong Wu, Tianyu Guo, Mikael Motelica-Heino, Lili Jiang

**Affiliations:** 1 Shandong Institute of Pomology, Tai’an, China; 2 Shandong Center of Technology Innovation for Modern Protected Fruit Tree, Tai’an, China; 3 College of Horticultural Science and Engineering, Shandong Agricultural University, Tai’an, Shandong, China; 4 Institute of Earth Sciences of Orleans, University of Orléans, Orléans, France

**Keywords:** *Fusarium verticillioides*, genome analysis, root rot, secondary metabolites, strawberry

## Abstract

Strawberry root rot is a serious soil-borne root disease with a high incidence rate that is extremely difficult to control. To identify the fungal pathogens responsible for strawberry root rot, diseased plants exhibiting root rot symptoms were collected from Jining, Shandong Province, China. Strain CMGF-RB13 was isolated from the roots of infected strawberry plants, and based on morphological characteristics and molecular analyses, was identified as *Fusarium verticillioides*. To our knowledge, this is the first study of strawberry root rot disease caused by *F. verticillioides* in China. The genome of this pathogenic fungal strain was sequenced using the Illumina MiSeq II and PacBio RS III platforms, and genome assembly analysis revealed a total length of 43,257,301 bp with a GC content of 47.89%. Analysis of potential secondary metabolites indicated that *F. verticillioides* CMGF-RB13 have potential biosynthetic capacity of fumonisin B1, choline, fusaric acid, oxyjavanicin, and bikaverin, and these are putative genomic predictions based on biosynthetic gene clusters, rather than experimentally confirmed metabolites. The genome sequence was presented primarily as a genomic resource supporting the characterization of the strawberry-associated isolate. The genomic information may serve as a basis for future diagnostic and disease-management studies.

## 1 Introduction

Strawberry (*Fragaria* × *ananassa* Duch.) is a perennial fruit and one of the most widely cultivated berry crops worldwide (Benashvili et al., 2025; Wu et al., 2025; Yang S. et al., 2025a), playing a vital role in the global agricultural economy (Chun et al., 2024; Chou et al., 2025; Sun et al., 2025; Xu et al., 2024; Yuan et al., 2025). The fruit is valued for its distinctive flavor and high content of beneficial phytochemicals (Jiang et al., 2025a; Lin et al., 2025; Negri et al., 2024; Yu et al., 2024; Zhang et al., 2025b). With the continued expansion of strawberry cultivation, global production was estimated at USD 14 billion in 2020 (Cai et al., 2025). China is the largest producer of strawberries and holds the largest cultivation area worldwide (Jiang et al., 2025b; Qian et al., 2025). However, prolonged continuous cropping, combined with the unique conditions of facility cultivation (Du et al., 2024), has intensified soil-borne diseases such as root rot, posing challenges to the development of the strawberry industry and causing significant economic losses for Chinese farmers (Lei et al., 2026).

Strawberry plants are susceptible to root rot during their growth cycle; this devastating soil-borne disease is particularly problematic in fields with continuous monocropping (Liu et al., 2024). The pathogens causing strawberry root rot are characterized by long incubation periods and complex pathogenic mechanisms. Root rot represents a more serious threat to strawberry production compared with other major strawberry diseases, such as anthracnose and gray mold. Infected plants typically exhibit decay in root and stem tissues, leading to significant losses in the strawberry industry (Zhang et al., 2025a,b). Continuous cultivation practices promote pathogen accumulation, resulting in frequent disease outbreaks and economic losses. Due to variations in soil properties, climatic conditions, and management practices, isolated pathogenic microorganisms differ across strawberry cultivation regions. Strawberry root rot is caused by infection with various pathogenic fungi throughout the entire growth period. It occurs worldwide and, in severe cases, can cause complete crop failure, thereby posing a major constraint on the development of the strawberry industry in China. Most root rot pathogens not only cause decay and necrosis of strawberry roots during infection but also induce necrosis at the root crown (Li et al., 2024; Ning et al., 2025). Studies have reported numerous pathogen types associated with strawberry root rot, with species of the *Fusarium* genus being the most prevalent, including *F. citri* (Liu A. et al., 2025; Liu B. et al., 2025), *F. asiaticum* (Xu J. et al., 2025; Xu Y. et al., 2025), *F. solani* (Yang et al., 2024a,b), *F. oxysporum* (Zhang et al., 2024), *F. cugenangense* (Shrestha et al., 2025), and *F. falciforme* (Payán-Arzapalo et al., 2024; Sun et al., 2024). *Fusarium verticillioides*, a hemibiotrophic pathogen (Wang M. et al., 2025), infects a range of plants, including safflower (Tirado-Ramírez et al., 2023), maize (Achimón and Pizzolitto, 2025; Xu J. et al., 2025; Xu Y. et al., 2025), rice (Liu A. et al., 2025; Liu B. et al., 2025), taoerqi (*Sinopodophyllum hexandrum*) (Wang et al., 2026), and tall fescue (Shi et al., 2026). However, *F. verticillioides* specifically and its genome levels had not been reported from Chinese strawberry root rot before.

Accurate identification of disease symptoms and causal pathogens is essential for effective prevention and control of strawberry diseases. In this study, diseased plants from a location were collected to characterize the pathogenic fungi responsible for strawberry root rot in Jining, Shandong province, China. They were used to isolate and purify the pathogen, which was subsequently subjected to pathogenicity assessment, morphological analysis, molecular identification, and genome analysis to provide a basis for diagnosing strawberry root rot.

## 2 Materials and methods

### 2.1 Sample collection and fungal isolation

In October 2025, approximately 50% of strawberry ‘Hongyan’ (*Fragaria* × *ananassa* ‘Benihoppe’) plants within a 0.05-ha site in Jining (116.99°E, 35.47°N), Shandong Province, China, exhibited root rot symptoms. Small tissue segments (1–2 mm) were excised from symptomatic areas of randomly selected strawberry roots. These samples were surface-sterilized with 75% ethanol for 30 s and 5% sodium hypochlorite (NaOCl) for 3 min, rinsed three times with sterile distilled water, dried using sterile paper towels, and plated onto 9-cm Petri dishes containing potato dextrose agar (PDA) (Tirado-Ramírez et al., 2023; Yan et al., 2025, 2026c).

### 2.2 Morphological identification

Microconidia and macroconidia were produced in 10-day-old cultures grown on carnation leaf agar medium (Tirado-Ramírez et al., 2023; Yang et al., 2024a,b). Spore-forming structures and conidial morphology were examined using an upright microscope (BX53F, Olympus, Japan).

### 2.3 Molecular identification

Genomic DNA was extracted from fungal isolates using the Ezup Column Fungi Genomic DNA Purification Kit (Sangon Biotech, Shanghai, China). The internal transcribed spacer (ITS) region and translation elongation factor 1-α (*TEF1-α*) were amplified using the primer pair ITS1/ITS2 (White et al., 1990) and EF1-728F/EF1-986R (Carbone and Kohn, 1999). The amplification products were purified and sequenced by Sangon Biotech, Shanghai, China.

### 2.4 Pathogenicity tests

Ten healthy strawberry ‘Hongyan’ plants were surface-sterilized using 75% ethanol and rinsed with sterile distilled water. Five plants were inoculated with a conidial suspension (10^6^ spores/mL) of the isolate, while five plants treated with sterile distilled water served as controls. The experiment was repeated twice. Following symptom development, the pathogen was re-isolated from lesions of the inoculated plants, thereby fulfilling Koch’s postulates (Tirado-Ramírez et al., 2023).

### 2.5 Whole-genome sequencing

Whole-genome sequencing of strain CMGF-RB13 was performed to characterize the pathogen at the genomic level. Fungal mycelia of CMGF-RB13 were cultured in potato dextrose broth (PDB) with shaking for 72 h prior to DNA extraction. The total amount of DNA was measured using a fluorescent dye (Quant-iT PicoGreen dsDNA Assay Kit), and DNA integrity was assessed via 1% agarose gel electrophoresis. The working DNA solution was stored at 4 °C, and the stock solution was stored at −20 °C.

Genomic DNA was fragmented using Covaris, purified, and sorted by size using magnetic beads. Using the combined 3′ → 5′ exonuclease and polymerase activities of the End Repair Mix, DNA fragments with protruding ends were repaired to generate blunt ends. A single adenine (A) base was then added to the 3′ end of each blunt DNA fragment, while the 3′ end of the adapter contained a complementary thymine (T) overhang, ensuring ligation of the DNA fragment to the adapter via A–T complementary base pairing and preventing intermolecular ligation between DNA fragments. The labeled adapter is subsequently incubated with the DNA fragments in the presence of ligase to facilitate their ligation. The ligation products were purified using magnetic beads to remove free and self-ligated adapter sequences. PCR was then performed to selectively enrich DNA fragments ligated at both ends, simultaneously amplifying the DNA library. The library was quantified using PicoGreen, and quality control of the PCR-enriched fragments was performed using an Agilent Bioanalyzer 2,100 to verify fragment size and distribution. Multiplexed DNA libraries were normalized to 10 nM and combined in equal volumes. The pooled libraries were then serially diluted from 10 nM to 4–5 pM before loading onto the sequencing instrument.

Prior to sequencing, quality control of the library was performed using an Agilent Bioanalyzer. A qualified library was characterized by a single peak with no adapter dimers. Library concentration was determined using the Quant-iT PicoGreen dsDNA Assay Kit on the Promega QuantiFluor fluorescence quantitative system and was required to exceed 2 nM. Qualified sequencing libraries containing non-repeating index sequences were serially diluted, pooled in proportions corresponding to the required sequencing volume, and denatured using NaOH into single strands before sequencing.

Genome sequencing was performed using the Illumina MiSeq II and PacBio RS III platforms (Shanghai Personal Biotechnology Co., Ltd). The sequencing data was finally assemble using Flye (v2.9.1). The integrity of the genome assembly was evaluated using BUSCO (Benchmarking Universal Single-Copy Orthologs, http://busco.ezlab.org, v5.4.5) and the scaffolds of the N50 and N90. Functional annotation of predicted genes was performed using the CARD,^1^ CAZy,^2^ DFVF,^3^ eggNOG,^4^ NR protein,^5^ and PHI databases,^6^ TCDB,^7^ as well as GO,^8^ KEGG,^9^ and secondary metabolite analyses (antiSMASH, https://antismash.secondarymetabolites.org) (Yang J. et al., 2025; Yan et al., 2026a,b; Zhang et al., 2025a,c).

### 2.6 Data analysis

Phylogenetic analyses of representative isolates based on concatenated ITS and *TEF1-α* sequences were conducted using PhyloSuite v2, and phylogenetic trees were constructed using the MrBayes algorithm (Zhao et al., 2025). Evolutionary models were tested using ModelFinder, and the best-fit substitution model for each gene was selected based on the Akaike information criterion corrected. Substitution models were selected by Partition Models and Bayesian Inference done by MrBayes 3.2.

## 3 Results

### 3.1 Root rot disease symptoms

In October 2025, approximately 50% of strawberry ‘Hongyan’ (*Fragaria* × *ananassa* ‘Benihoppe’) plants within a 0.05-ha site in Jining, Shandong province, China, exhibited root rot symptoms, including chlorosis, necrosis and rot of vascular bundles, stunted growth, and reflexed plants bent toward the ground (Figure 1).

**Figure 1:**
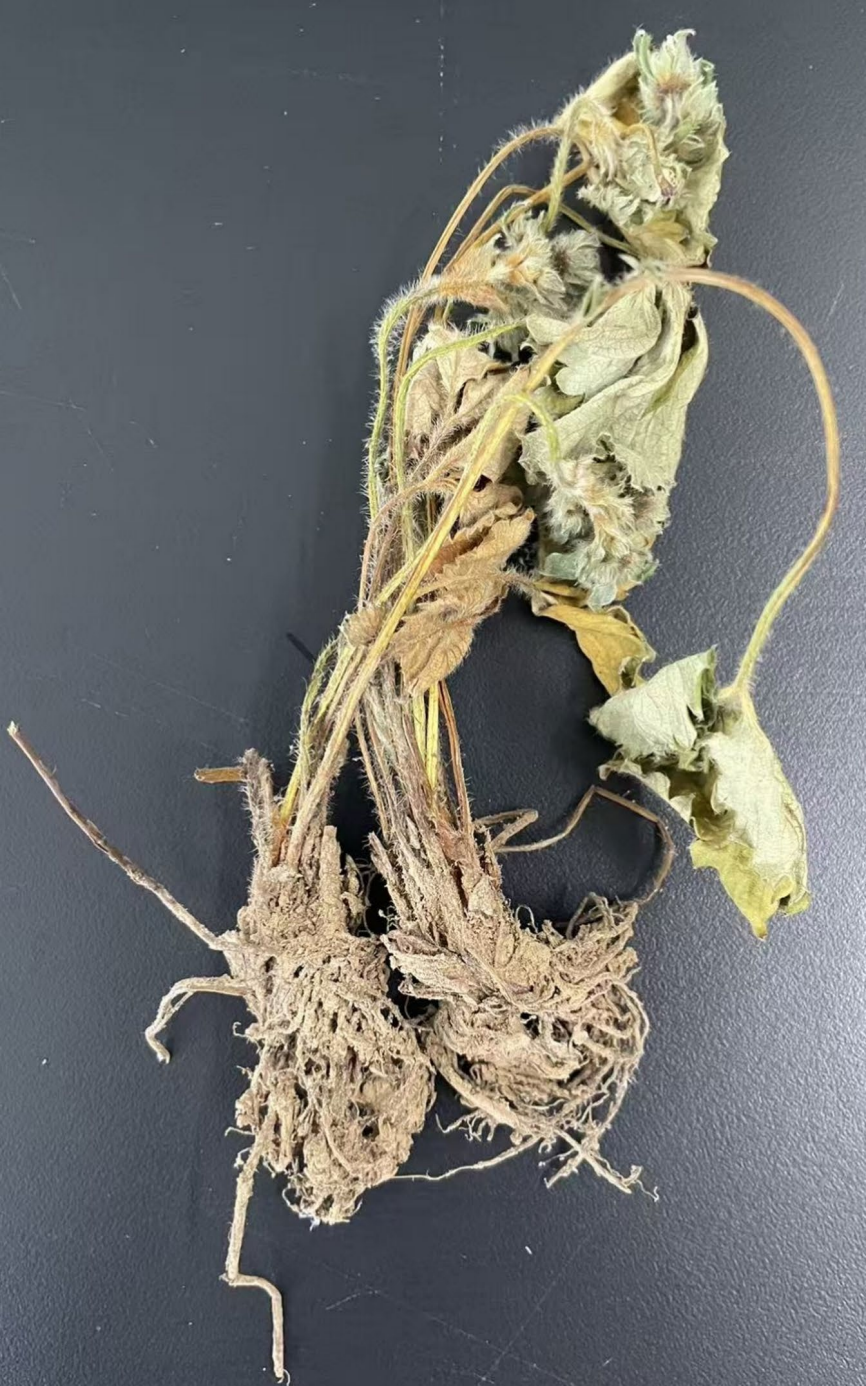
Typical symptoms of root rot in collected strawberry plant.

### 3.2 Morphology and pathogen verification of strain CMGF-RB13

Finally, five fungal isolates with similar cultural and morphological characteristics were consistently isolated from symptomatic tissues through single-spore culture on PDA. A representative fungal isolate, designated CMGF-RB13, was randomly selected and cultured on PDA plates for 5 days (Figures 2A,B). Microconidia were hyaline, with zero to two septa, were oval or ellipsoidal in shape, and measured 9.03 (4.5–14.7) × 3.07 (1.5–4.2 μm) (*n* = 30) (Figure 2C). Macroconidia were hyaline, slightly curved, with three to five septa, and measured 23.5 (16–72) × 3.03 (0.9–6.6) μm (*n* = 30) (Figure 2D). It is the formation of long chains of microconidia produced by monophialides. Based on these morphological characteristics, the isolates were identified as *Fusarium* spp. (Tirado-Ramírez et al., 2023).

**Figure 2:**
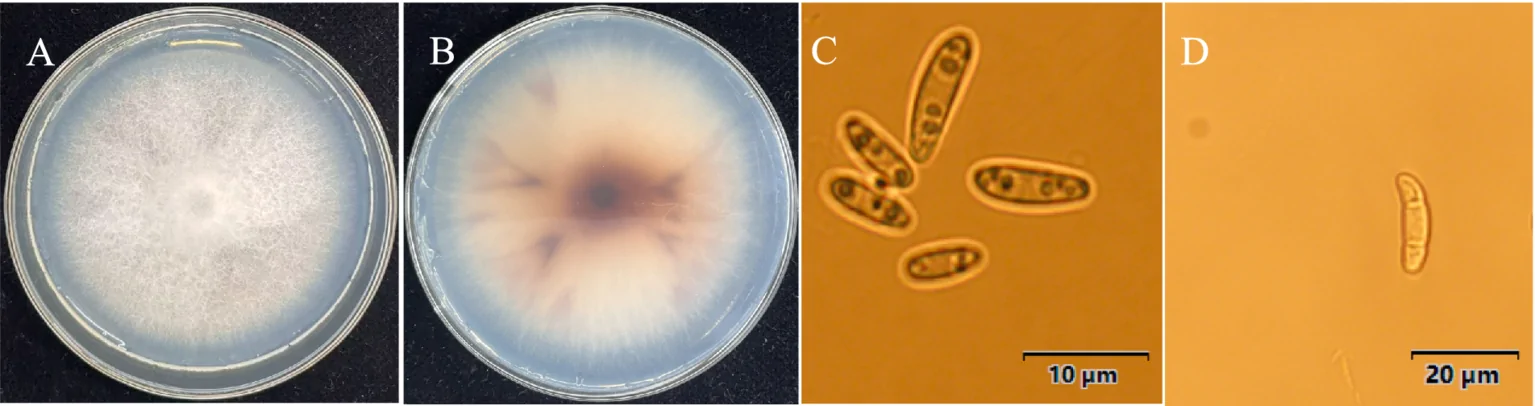
Front **(A)** and back **(B)** views of fungal CMGF-RB13 colonies isolated from infected strawberry plant, and conidial morphology **(C,D)** of CMGF-RB13.

### 3.3 Phylogenetic analysis of strain CMGF-RB13

A phylogenetic tree constructed from ITS (PZ441170) and *TEF1-α* (PZ577210) gene sequences revealed that CMGF-RB13 is most closely related to *F. verticillioides*, and Bayesian posterior probability is 1 (Figure 3). Rotting and necrosis were observed in the roots of inoculated plants (Figure 4). The pathogen was re-isolated from all symptomatic plants, displayed the same morphological and molecular traits as the initial isolate and was again identified as *F. verticillioides*, thereby confirming Koch’s postulates.

**Figure 3:**
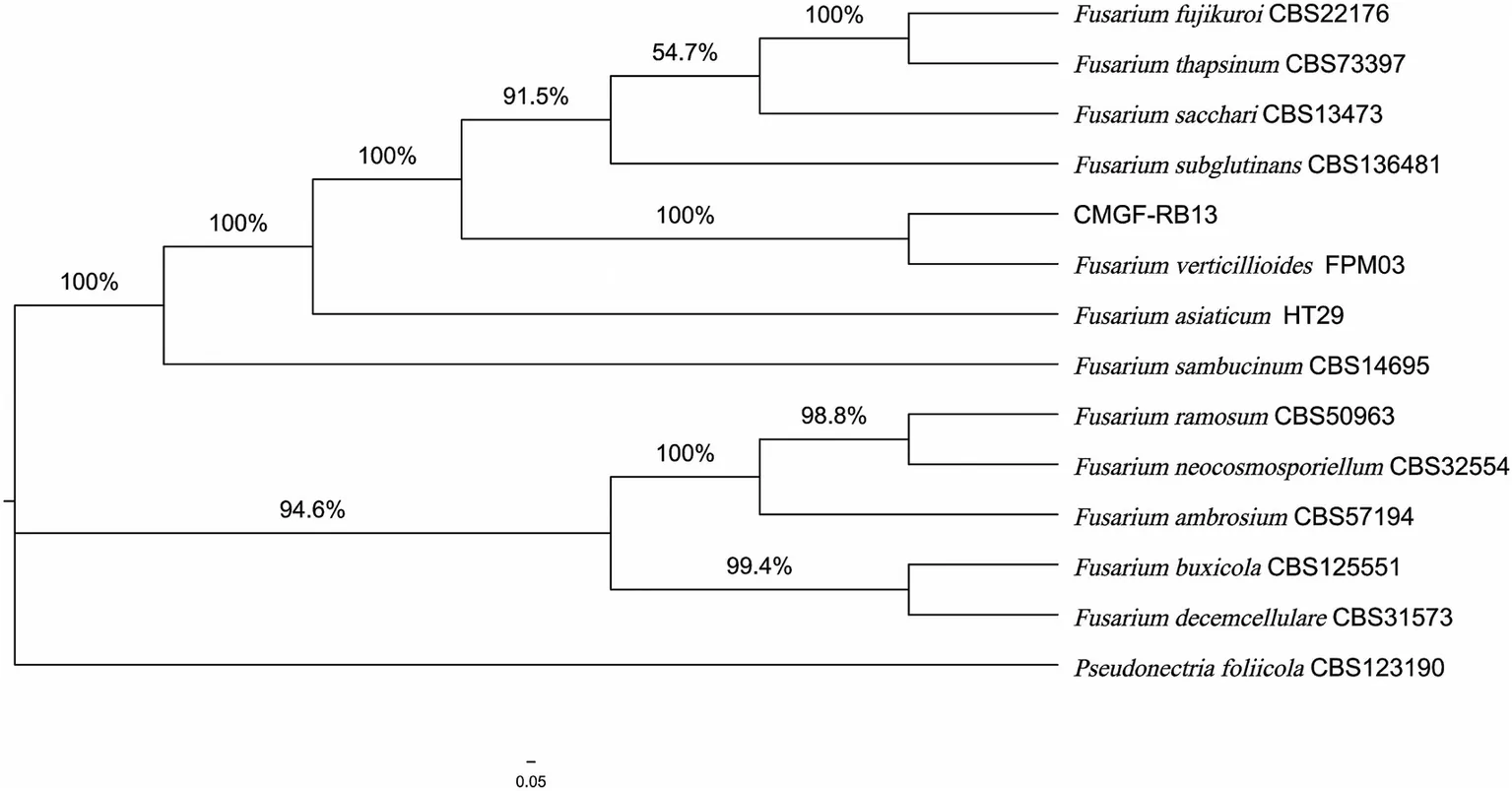
Phylogenetic tree of isolate CMGF-RB13 based on concatenated ITS and *TEF1-α* sequences. Markov Chain Monte Carlo (MCMC) settings included 20,000 generations, a burn-in fraction of 0.25, 1,000 sampling frequency, the number of independent runs of 2, and the number of independent chains of 4. Posterior Probability (≥0.54) is shown above the branches.

**Figure 4:**
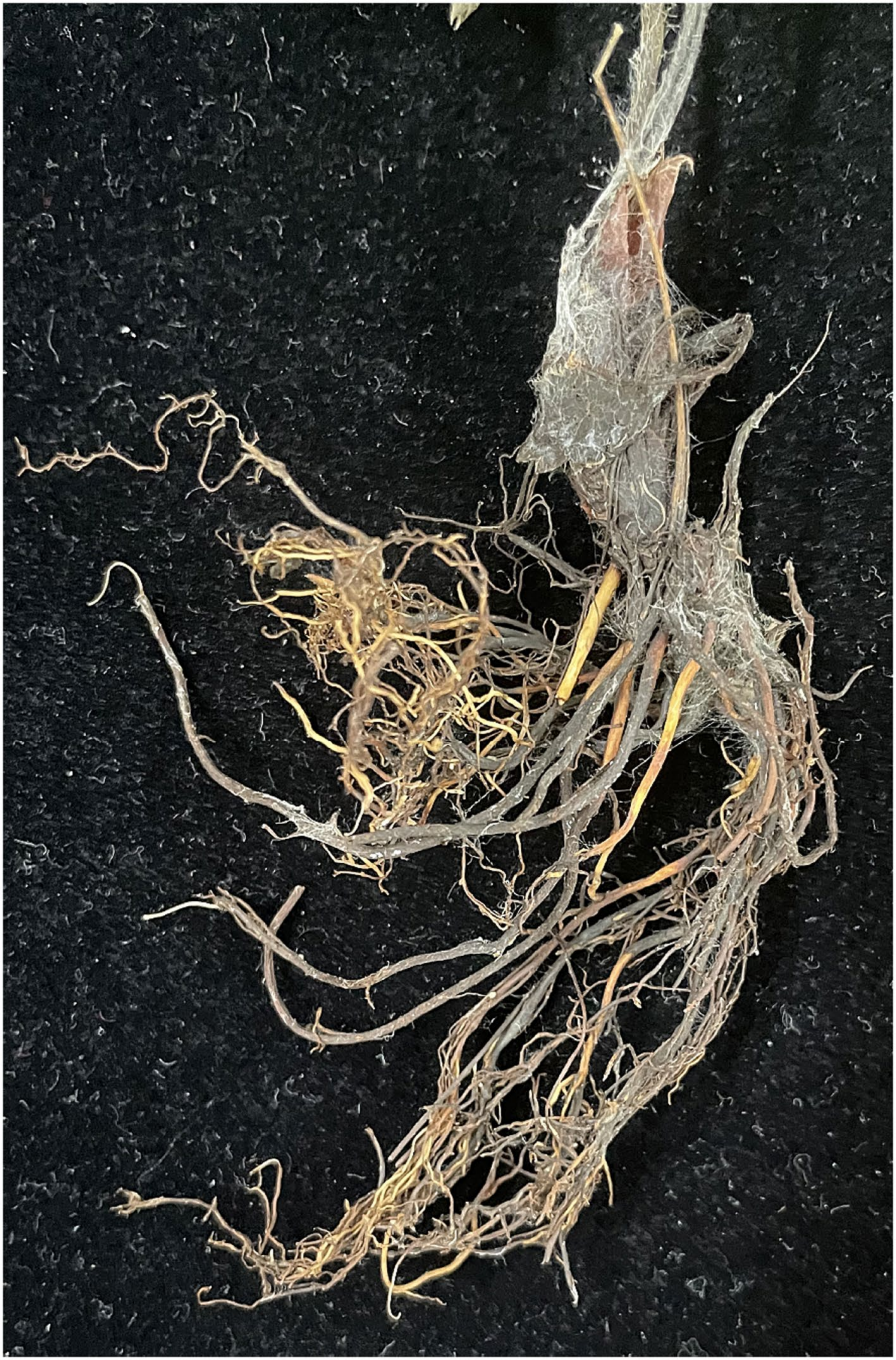
Symptoms of strawberry plant following inoculation with CMGF-RB13.

### 3.4 Results of genome sequencing and assembly

The sequencing data was finally assemble using Flye (v2.9.1). The assembled genome of *F. verticillioides* CMGF-RB13 (Genome submission JBYLFF000000000) has a total length of 43,257,301 bp and a GC content of 47.89% (Figure 5A). The copy numbers of ncRNA, 5S rRNA, 5.8S rRNA, 18S rRNA, 28S rRNA, and tRNA were 46, 59, 3, 3, 4, and 295, respectively, with total lengths of 7,167, 6,838, 456, 4,199, 11,754, and 25,984 bp, respectively. The scaffolds of the N50 and N90 were 4,306,550 bp and 2,502,284 bp, respectively. A total of 4,485 complete BUSCOs were identified, representing 99.8% of all BUSCOs. Functional annotation of protein-coding genes was conducted using multiple databases and analyses, including the Comprehensive Antibiotic Resistance Database (CARD; 5), the Carbohydrate-Active Enzymes Database (CAZy; 792), the Database of Fungal Virulence Factors (DFVF; 1,418), Gene Ontology (GO) analysis (8,716), Kyoto Encyclopedia of Genes and Genomes (KEGG) analysis (4,217), KOG (13,162), MEROPS (6,779), the Non-Redundant (NR) database (14,082), P450 (13,799), Pfam (10,211), the Pathogen–Host Interactions (PHI) database (3,787), Secretory (1,197), Signal (1,448), SwissProt (9,210), T3SS (5,139), TargetP (14,121), the Transporter Classification Database (TCDB; 2,177), and TMHMM (2,812) (Figure 5B).

**Figure 5:**
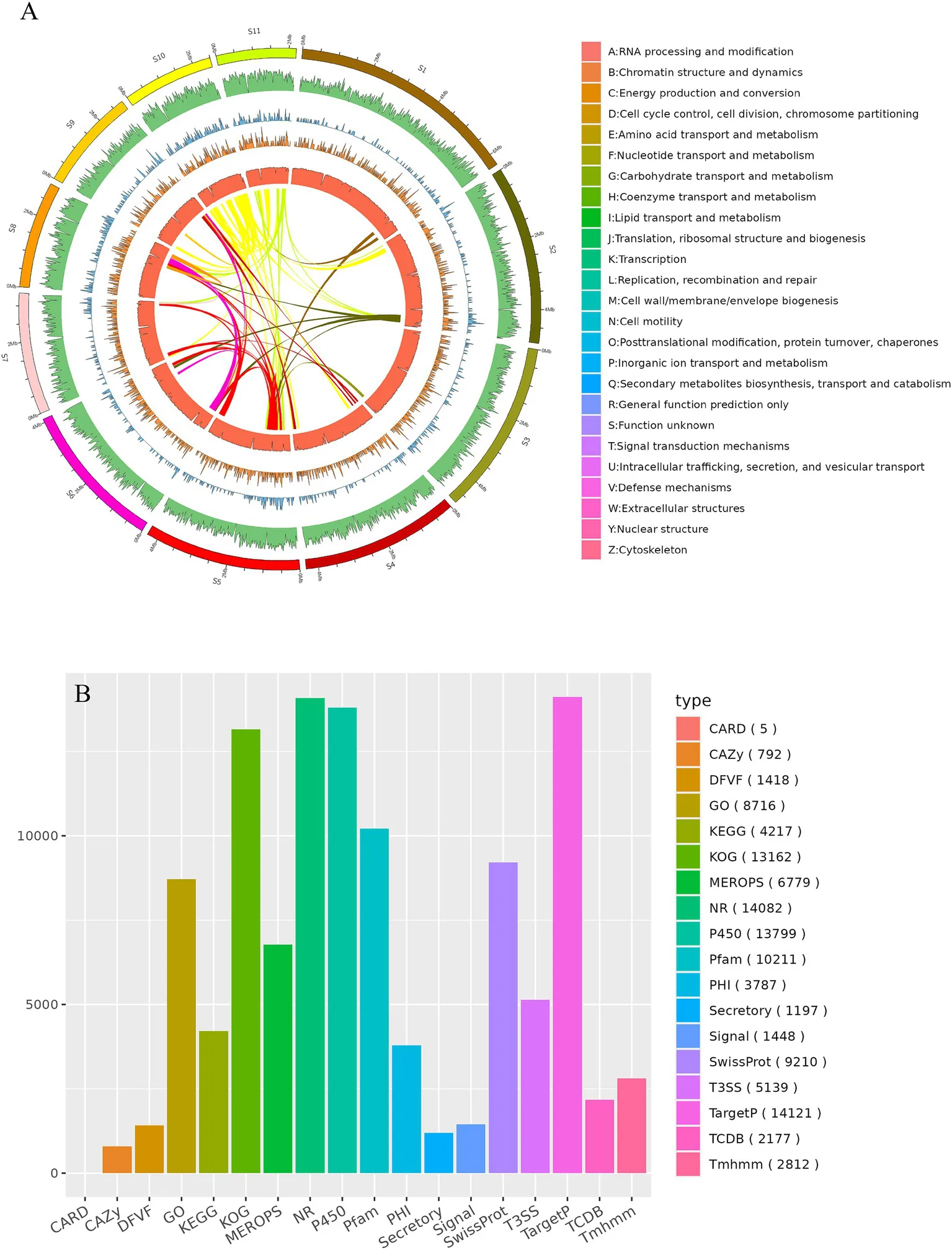
Genomic circle map of CMGF-RB13 **(A)**, and overview of protein-coding gene functional annotation in different databases **(B)**.

### 3.5 CARD analysis

CARD analysis identified five antibiotic resistance genes, accounting for 0.035% of annotated genes; however, no antibiotic target or antibiotic biosynthesis genes were detected (Table 1).

**Table 1:** Antibiotic resistance analysis statistics in CARD.

Property	Number of genes	Percentage (%)	Query_name
Antibiotic resistance	5	0.035	scaffold1.t699 scaffold11.t210 scaffold11.t407 scaffold2.t1025 scaffold3.t472
Antibiotic target	0	0	–
Antibiotic biosynthesis	0	0	–

### 3.6 CAZyme analysis

Analysis using the CAZy database (Figure 6) identified 107 glycosyl transferases (GTs), 24 polysaccharide lyases (PLs), 180 carbohydrate esterases (CEs), 125 auxiliary activity enzymes (AAs), 31 carbohydrate-binding modules (CBMs), and 325 glycoside hydrolases (GHs).

**Figure 6:**
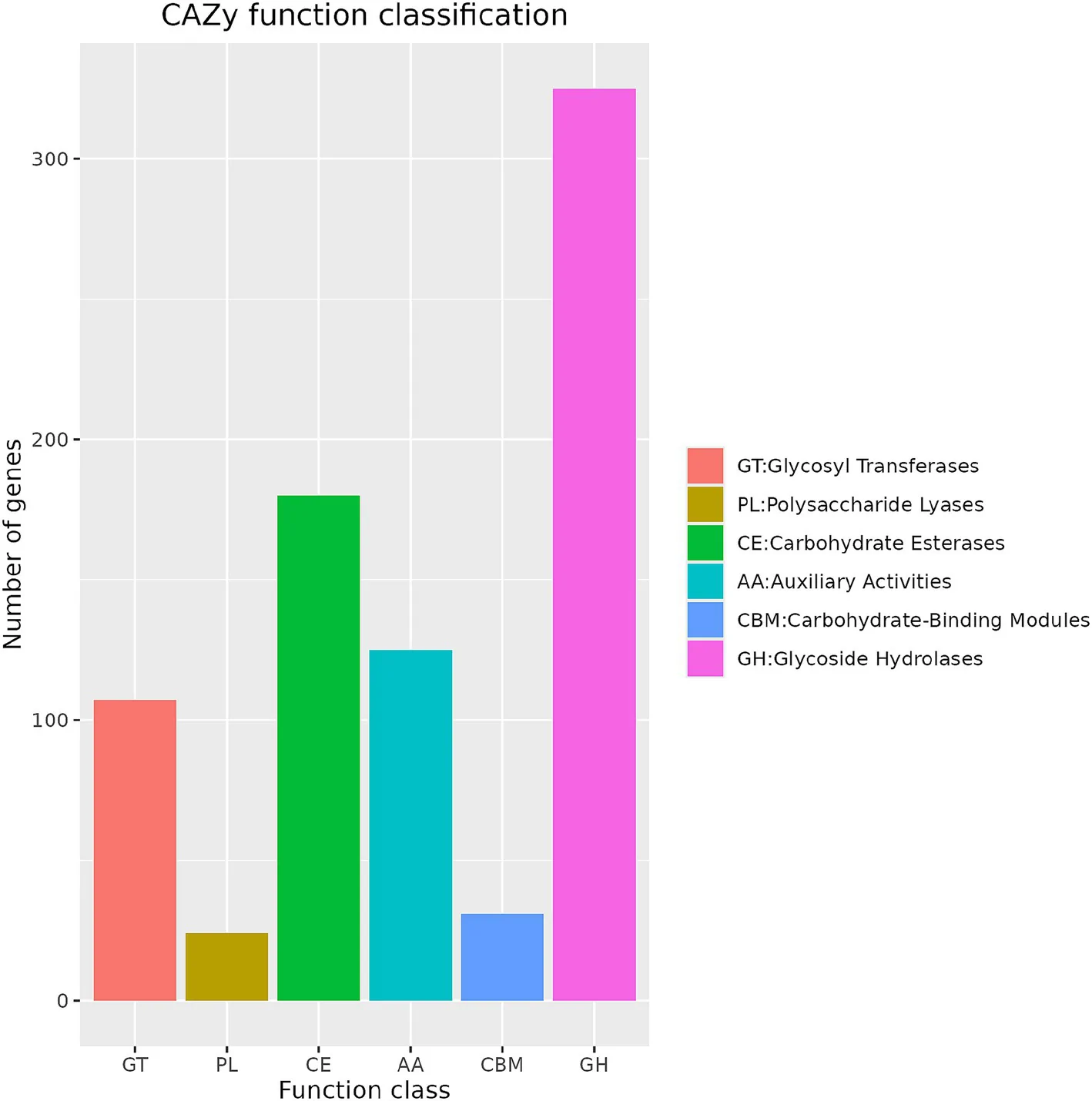
CAZy function classification profile.

### 3.7 GO analysis

GO analysis indicated that *F. verticillioides* CMGF-RB13 is associated with three distinct classification schemes: biological processes, molecular functions, and cellular components (Figure 7). The predictive annotations included “mycotoxin metabolic process (GO:0043385),” “mycotoxin biosynthetic process (GO:0043386),” “toxin biosynthetic process (GO:0009403),” “toxin metabolic process (GO:0009404),” and “aflatoxin biosynthetic process (GO:0045122)” (Table 2).

**Figure 7:**
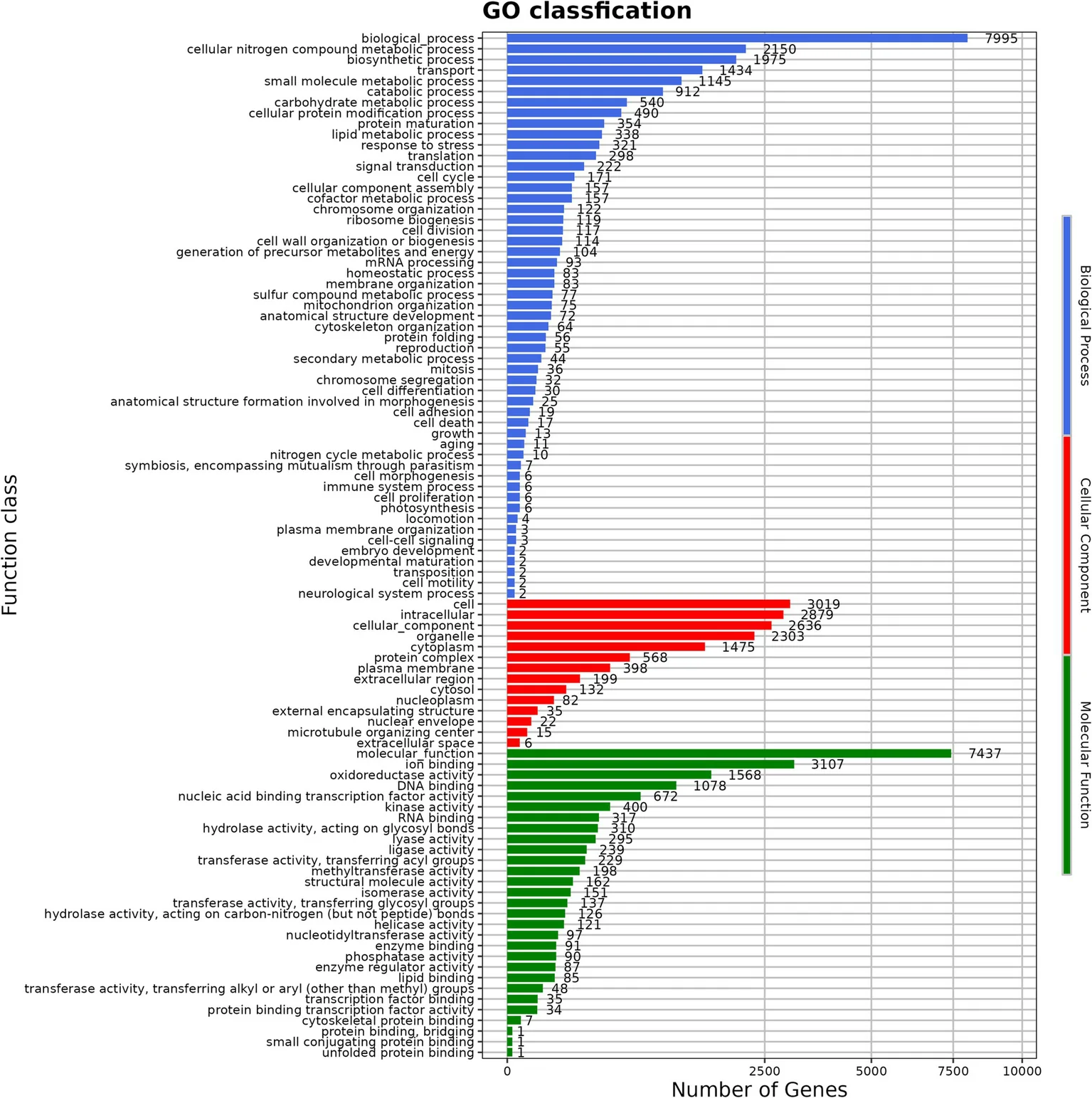
GO function classification profile.

**Table 2:** Toxin-related processes in GO.

protein_id	GO_number	GO_function	GO_ontologies
scaffold3.t1303	GO:0046222	aflatoxin metabolic process	Biological process
scaffold3.t1303	GO:0043385	mycotoxin metabolic process	Biological process
scaffold3.t1303	GO:0043386	mycotoxin biosynthetic process	Biological process
scaffold3.t1303	GO:0009403	toxin biosynthetic process	Biological process
scaffold3.t1303	GO:0009404	toxin metabolic process	Biological process
scaffold3.t1303	GO:0045122	aflatoxin biosynthetic process	Biological process
scaffold4.t128	GO:0009403	toxin biosynthetic process	Biological process
scaffold4.t128	GO:0009404	toxin metabolic process	Biological process

### 3.8 KEGG analysis

KEGG analysis (Figure 8) annotated 2,379 genes of *F. verticillioides* CMGF-RB13 to “Protein families: genetic information processing” within Brite hierarchies, 385 genes to “Transport and catabolism” under “Cellular Processes,” 580 genes to “Signal transduction” under “Environmental Information Processing,” 334 genes to “Translation” under “Genetic Information Processing,” 587 genes to “Amino acid metabolism” under “Metabolism,” 209 genes to “Unclassified: metabolism” in “Unclassified: metabolism,” and 307 genes to “Endocrine system” under “Organismal Systems.”

**Figure 8:**
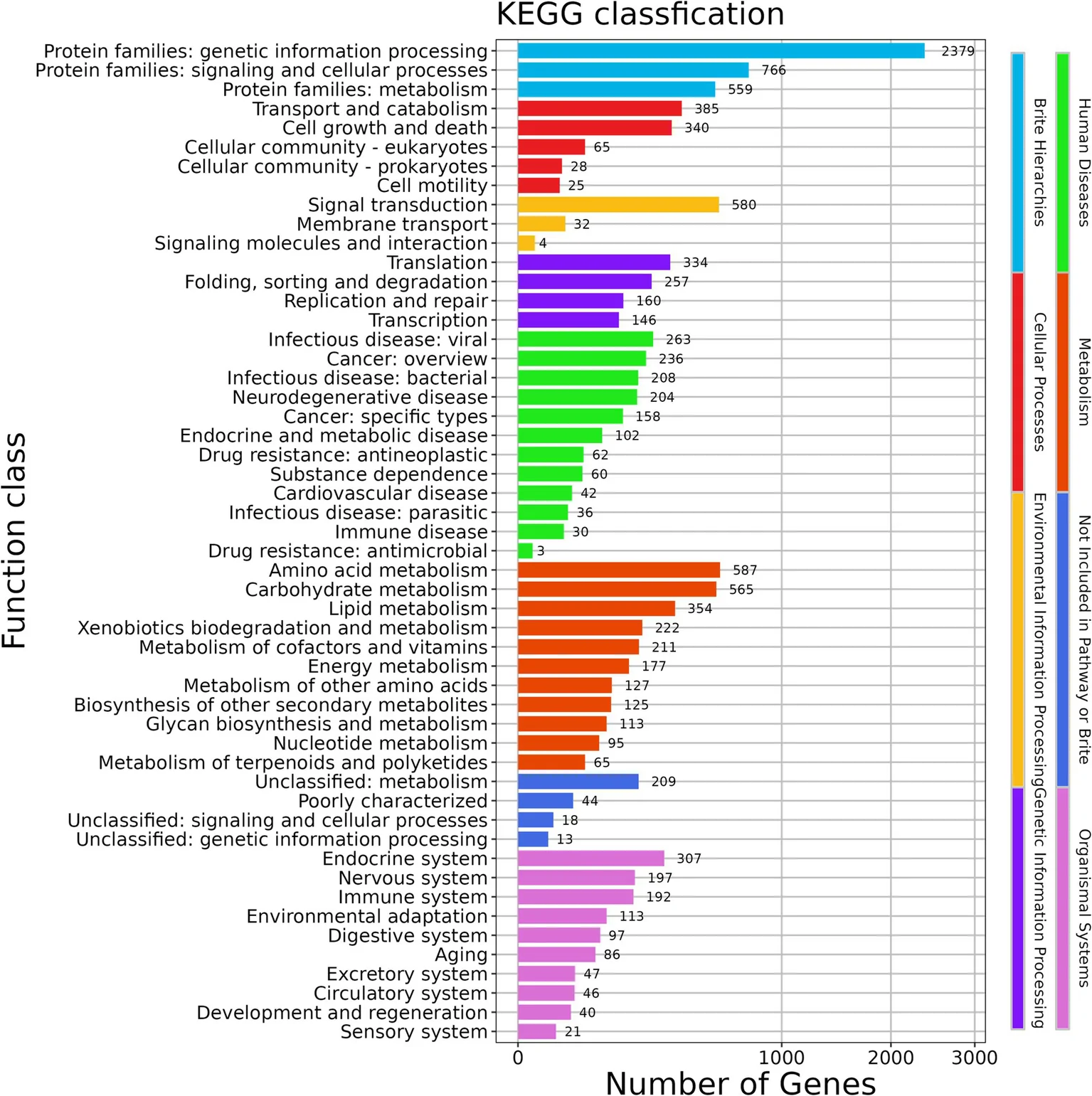
KEGG function classification profile.

### 3.9 Evolutionary genealogy of genes: non-supervised orthologous groups (eggNOG) analysis

The largest category comprised open reading frames (ORFs) of unknown function, totaling 6,449 and accounting for 49.00% of all annotated genes. This finding indicates that the functional annotation of *F. verticillioides* CMGF-RB13 remains incomplete and requires further investigation (Figure 9). eggNOG analysis showed that ORFs were distributed across various functional categories, including “RNA processing and modification (288),” “Chromatin structure and dynamics (81),” “Energy production and conversion (480),” “Cell cycle control, cell division, chromosome partitioning (113),” “Amino acid transport and metabolism (576),” “Nucleotide transport and metabolism (130),” “Carbohydrate transport and metabolism (996),” “Coenzyme transport and metabolism (116),” “Lipid transport and metabolism (371),” “Translation, ribosomal structure, and biogenesis (392),” “Transcription (396),” “Replication, recombination, and repair (209),” “Cell wall/membrane/envelope biogenesis (130),” “Cell motility (2),” “Posttranslational modification, protein turnover, chaperones (590),” “Inorganic ion transport and metabolism (239),” “Secondary metabolites biosynthesis, transport, and catabolism (680),” “General function prediction only (0),” “Signal transduction mechanisms (380),” “Intracellular trafficking, secretion, and vesicular transport (351),” “Defense mechanisms (90),” “Extracellular structures (4),” “Nuclear structure (4),” and “Cytoskeleton (95).”

**Figure 9:**
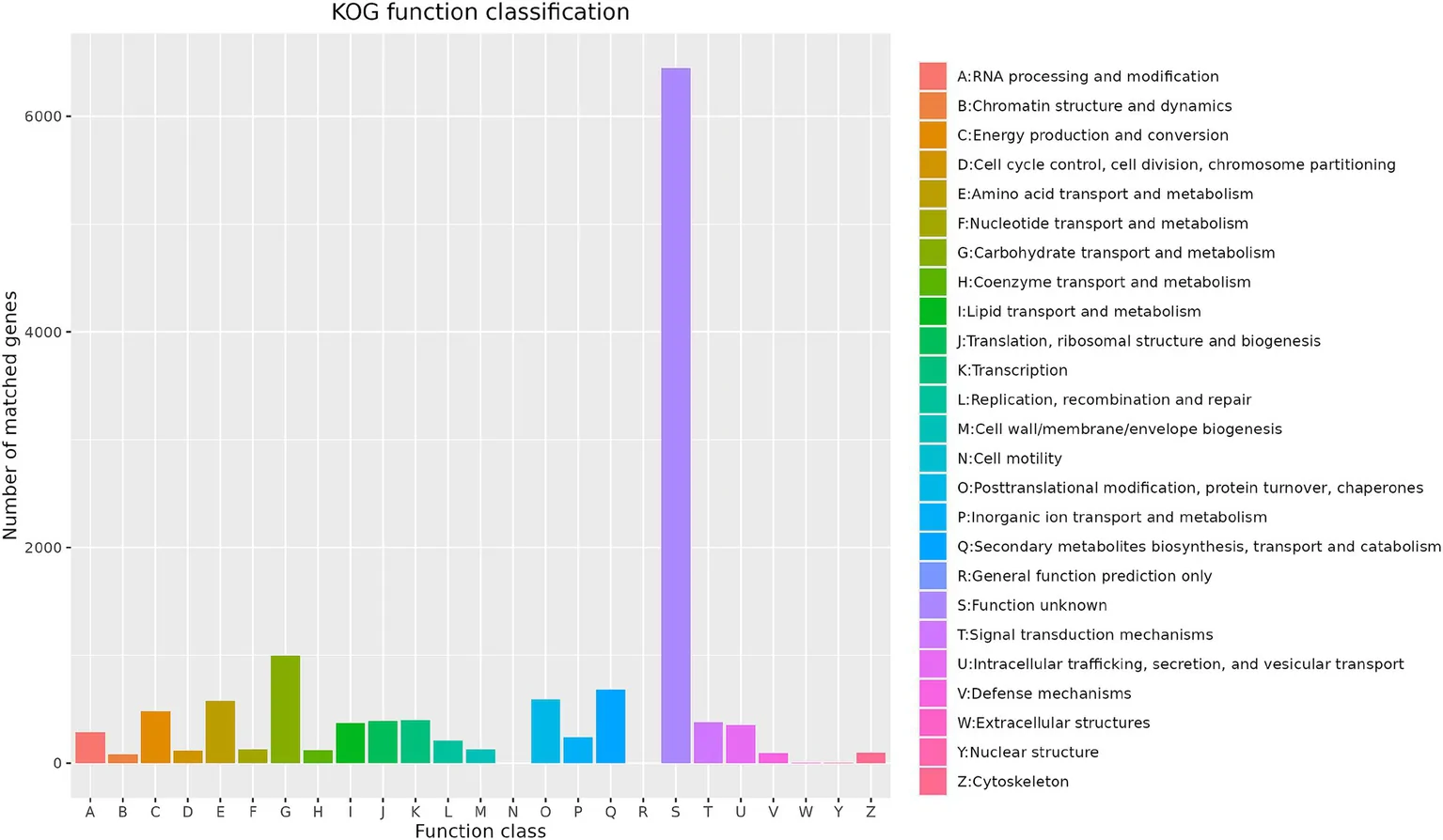
eggNOG function classification profile.

### 3.10 PHI-base analysis

PHI-base annotations were described as homologs of genes previously associated with pathogenicity or virulence in other organisms. PHI-base analysis identified 255 homologous genes associated with loss of pathogenicity, 1,865 homologous genes with no effect on pathogenicity, 1,713 homologous genes with reduced virulence, and 167 homologous genes with increased pathogenicity (hypervirulence) (Figure 10).

**Figure 10:**
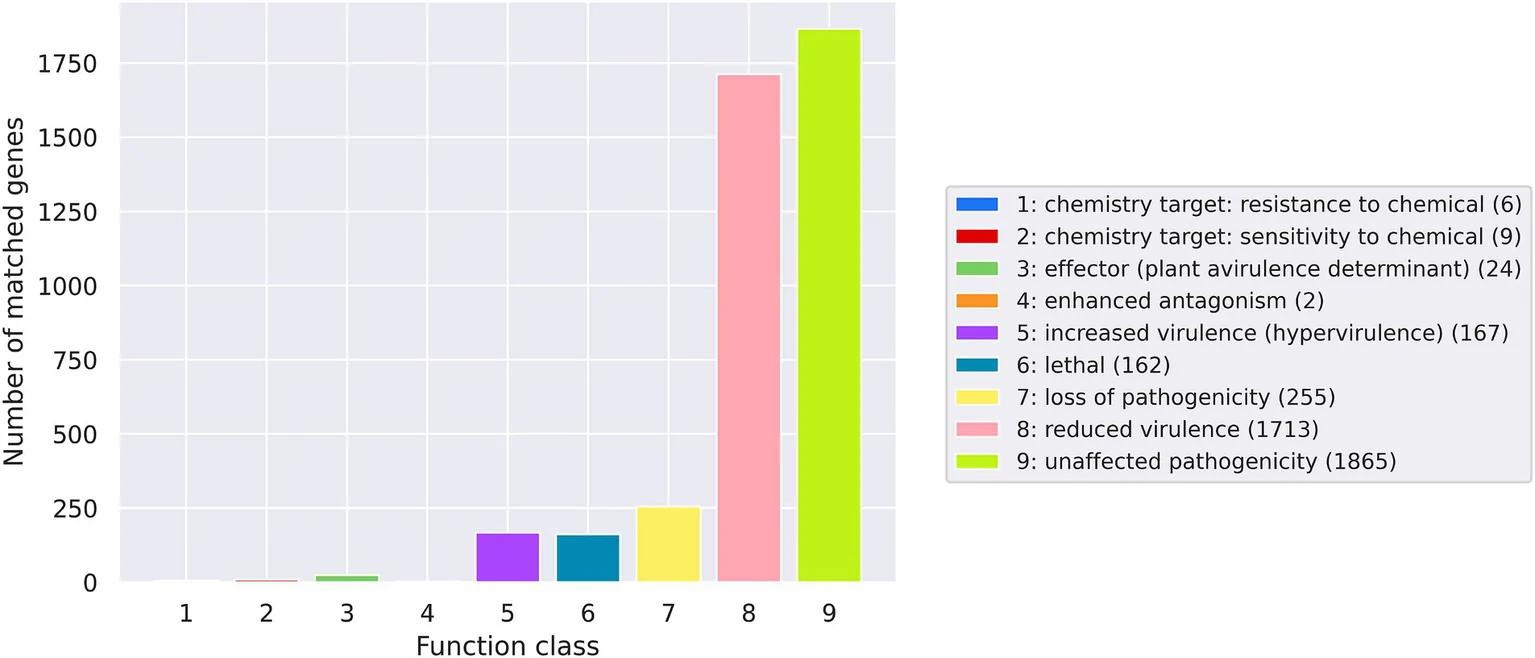
PHI function classification profile.

### 3.11 TCDB analysis

TCDB primarily provides functional classification of transporter proteins. TCDB analysis identified 899 genes in the “Electrochemical potential-driven transporters” category within the primary classification (Figure 11A) and 893 genes in the “Porters (uniporters, symporters, antiporters)” category within the secondary classification (Figure 11B). These annotations indicate that the corresponding genes are closely associated with the predicted transporter functions in *F. verticillioides* CMGF-RB13.

**Figure 11:**
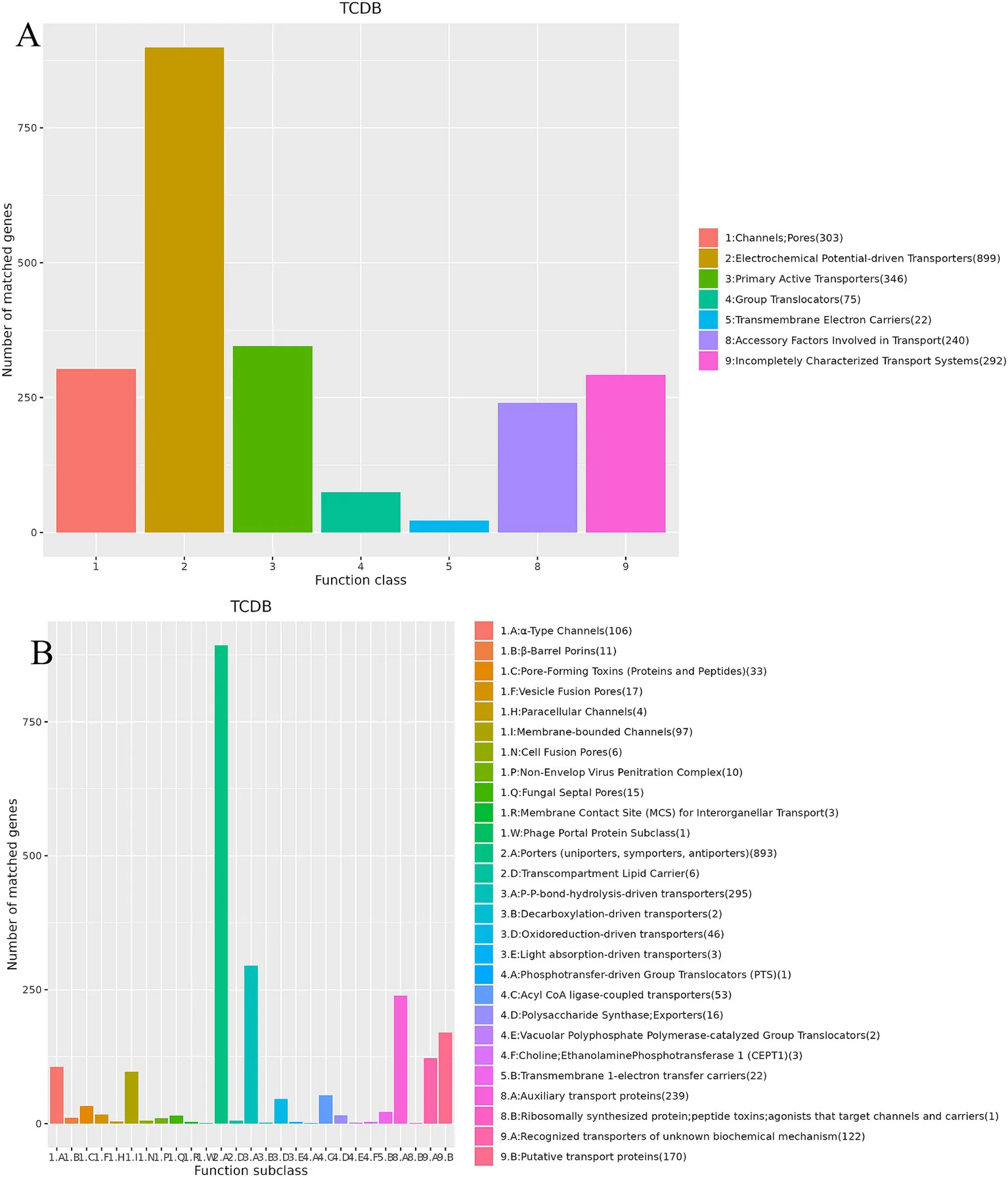
TCDB annotation species statistics chart. **(A)** Primary classification; **(B)** Secondary classification.

### 3.12 NR analysis

NR analysis (Figure 12) showed that strain CMGF-RB13 shared the highest sequence similarity (99.86%) with members of the genus *Fusarium*. Additionally, protein-coding gene sequences exhibited similarity to proteins from *Ditylenchus*, *Gibberella*, *Zea*, and *Lupinus*, with alignment lengths of 9, 5, 3, and 3 amino acids, respectively. Notably, four fumonisin-related genes (scaffold1.t1405, scaffold1.t1410, scaffold1.t1413, and scaffold1.t1418) were identified (Table 3).

**Figure 12:**
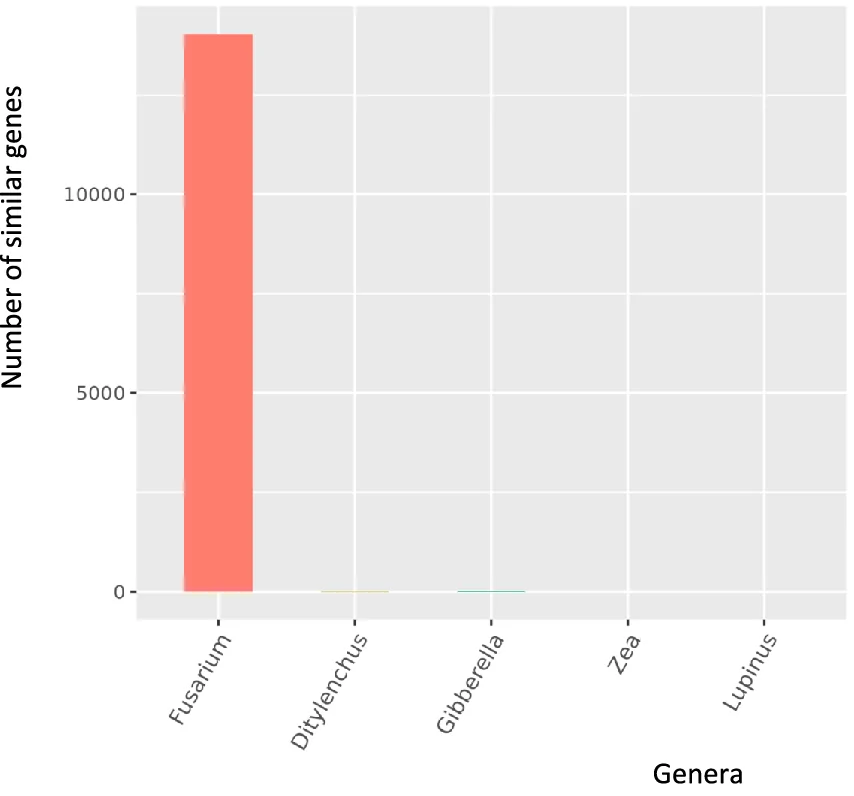
NR function classification profile.

**Table 3:** Fumonisin biosynthesis processes in NR.

Query_name	Hit_length	Hit_start	Hit_end	Aln_length	Identity	*E* value
scaffold1.t1405	1,489	2	1,489	1,488	94.83	0
scaffold1.t1410	548	1	548	563	95.56	0
scaffold1.t1413	552	1	492	492	99.80	0
scaffold1.t1418	2,586	1	2,586	2,586	99.81	0

### 3.13 Analysis of predicted secondary metabolites

Analysis of potential secondary metabolites predicted that *F. verticillioides* CMGF-RB13 have potential biosynthetic capacity of fumonisin B1, choline, fusaric acid, oxyjavanicin, and bikaverin (Table 4). However, the results were not experimentally confirmed compounds.

**Table 4:** Analysis of predicted secondary metabolites.

Region	Type	From	To	Most similar known cluster	Similarity
Region1.4	T1PKS	5,437,857	5,501,835	fumonisin B1 Polyketide	87%
Region2.2	NRPS-like	419,949	483,001	choline NRP	100%
Region2.6	NRPS, T1PKS	4,536,809	4,609,085	equisetin NRP + Polyketide	45%
Region2.7	NRPS-like, T1PKS	4,792,199	4,882,969	fusaric acid Polyketide	90%
Region3.2	terpene	3,387,006	3,416,626	squalestatin S1 Terpene	40%
Region3.4	T1PKS	4,047,656	4,114,719	oxyjavanicin Polyketide	100%
Region4.1	NRPS-like	1	60,686	fusaridione A;(3R,5S)-5-(4-hydroxybenzyl)-3-methyl-3-((2E,4E,6E,8E,10E)-4,8,10-trimethyldodeca-2,4,6,8,10-pentaenoyl)pyrrolidine-2,4-dione NRP + Polyketide	18%
Region4.2	T1PKS	80,781	147,057	bikaverin Polyketide	85%
Region6.1	NRPS	15,621	72,137	aspirochlorine NRP	13%
Region6.5	T1PKS	3,416,983	3,484,018	depudecin Polyketide: Iterative type I polyketide	100%
Region7.1	NRPS, NRPS-like,fungal-RiPP-like	221	134,013	peramine NRP	100%
Region8.1	fungal-RiPP-like	1,982,078	2,068,920	lucilactaene Polyketide	23%
Region8.2	terpene	2,165,889	2,197,595	koraiol Terpene	100%
Region9.1	NRPS, T1PKS	308,150	380,009	lucilactaene Polyketide	61%
Region10.1	T1PKS,terpene	283,239	349,121	acorenol Terpene	100%
Region10.5	T1PKS	2,008,705	2,076,427	trichoxide Polyketide	91%
Region11.2	NRPS, T1PKS,terpene	1,840,877	1,959,470	gibepyrone-A Polyketide	80%
Region11.3	T1PKS	2,004,646	2,072,256	fujikurin A; fujikurin B; fujikurin C; fujikurin D Polyketide	66%
Region12.2	NRPS-like	662,344	721,671	HC-toxin NRP	100%

## 4 Discussion

*Fusarium verticillioides* has multiple routes of infection, including systemic infection in seedlings, entry through the stigma, and infection of stems and ears via mechanical damage (Estrada et al., 2024). Consequently, diseases caused by this fungus result in substantial yield reductions and economic losses worldwide and require serious attention (Sui et al., 2026). The present study demonstrates that *F. verticillioides* can cause root rot disease in strawberries in Jining, China. However, the pathogenicity assessment remains limited by the small sample size and the absence of quantitative disease severity measurements. Future studies should focus on disease management strategies on the basis of these data, including biological, chemical, and physical control approaches, to prevent further localized outbreaks (He et al., 2023; Jambhulkar et al., 2022; Yan et al., 2021).

Genomic approaches are important tools for studying plant pathogenic fungi such as *F. verticillioides* (Estrada et al., 2024; Narváez-Zapata et al., 2025; Li et al., 2025). *De novo* assembly from Illumina NovaSeq 6,000 sequencing data yielded 456 contigs, comprising a 42.8-Mb genome assembly with a GC content of 48.26% for *F. verticillioides* REC01 (Estrada et al., 2024). The assembled genome of *F. verticillioides* DA42 comprised 175 contigs (=200 bp) totaling 42.27 Mb, with a GC content of 48.51% (Narváez-Zapata et al., 2025). In addition, a chromosomal-level gold-standard assembly of *F. verticillioides* FvSZ22 revealed nuclear and mitochondrial genome sizes of 43.33 Mb and 53.73 kb, respectively (Li et al., 2025). In the present study, the *F. verticillioides* CMGF-RB13 genome has a total length of 43,257,301 bp, with a GC content of 47.89%. These findings provide a foundation for further studies on strawberries, such as investigating the genetic diversity of pathogen populations, monitoring resistance to chemical pesticides, and developing specific molecular diagnostic methods. The genome sequence is presented primarily as a genomic resource to support the characterization of a strawberry-associated isolate, not as a source of novel genomic discoveries. PHI-base analysis identified 255 genes associated with loss of pathogenicity, 1,865 genes with no effect on pathogenicity, 1,713 genes with reduced virulence, and 167 genes with increased pathogenicity (hypervirulence). Therefore, the PHI-base findings are relevant to pathogenic mechanisms. Moreover, comparative genomic or phylogenomic analysis with representative *F. verticillioides* isolates from different hosts would substantially help determine whether CMGF-RB13 has distinctive genomic characteristics.

*Fusarium verticillioides* is capable of producing significant amounts of mycotoxins, collectively known as *Fusarium* mycotoxins, which include trichothecenes, zearalenone, and fumonisins (Yang W. et al., 2025). Fumonisins are sphingosine-like mycotoxins associated with various toxic effects, including neurotoxicity, immunotoxicity, and carcinogenicity, through their action on sphingolipid metabolism, oxidative stress, and apoptosis pathways. *Fusarium verticillioides* is the predominant fumonisin-producing phytopathogen in plants (Wang L. et al., 2025). Fumonisins are classified into fumonisin B (FB) or fumonisin C (FC) groups depending on the presence (FBs) or absence (FCs) of a terminal methyl or hydroxyl group (Li et al., 2025). FBs structurally resemble sphinganine, a key intermediate in ceramide biosynthesis, and competitively inhibit ceramide synthase, thereby disrupting sphingolipid metabolism (Li et al., 2025; Jacquat et al., 2025). The presence of mycotoxins in plants adversely impacts human and animal health, leading to economic losses and affecting international trade relations (Estrada et al., 2024). Analysis of potential secondary metabolites predicted that *F. verticillioides* CMGF-RB13 have potential biosynthetic capacity of fumonisin B1, choline, fusaric acid, oxyjavanicin, and bikaverin base on the identification of biosynthetic gene clusters, but does not experimentally demonstrate actual metabolite production. Morever, several biosynthetic gene cluster predictions are based on very low similarity scores including aspirochlorine at 13%, fusaridione at 18%, equisetin at 45%, and squalestatin S1 at 40%. Experimental validation is necessary to confirm any of these metabolites. To further validate these toxin-related mechanisms, future studies will employ integrated metabolomic and transcriptomic analyses to elucidate the precise pathogenic strategies of strain CMGF-RB13.

In conclusion, *Fusarium verticillioides* CMGF-RB13, isolated from infected strawberry plants and identified based on conidial morphology and molecular analyses, was confirmed to cause strawberry root rot. The main novelty appears to be the first confirmed occurrence of *F. verticillioides* causing strawberry root rot in China. Genome assembly revealed that *F. verticillioides* CMGF-RB13 has a total length of 43,257,301 bp with a GC content of 47.89%. Functional annotation of protein-coding genes identified matches across multiple databases, including CARD (5), CAZy (792), DFVF (1,418), GO (8,716), KEGG (4,217), KOG (13,162), MEROPS (6,779), NR (14,082), P450 (13,799), Pfam (10,211), PHI (3,787), Secretory (1,197), Signal (1,448), SwissProt (9,210), T3SS (5,139), TargetP (14,121), TCDB (2,177), and TMHMM (2,812). The genome sequence should be presented primarily as a genomic resource supporting the characterization of the strawberry-associated isolate. Furthermore, analysis of potential secondary metabolites indicated that *F. verticillioides* CMGF-RB13 have potential biosynthetic capacity of fumonisin B1, choline, fusaric acid, oxyjavanicin, and bikaverin, and these are putative genomic predictions based on biosynthetic gene clusters, rather than experimentally confirmed metabolites.

## Data Availability

The datasets presented in this study can be found in online repositories. The names of the repository/repositories and accession number(s) can be found in the article/supplementary material.

## Footnotes

1
https://card.mcmaster.ca/

2
http://www.cazy.org

3
http://sysbio.unl.edu/DFVF/

4
http://eggnogdb.embl.de/

5
http://ftp.ncbi.nlm.nih.gov/blast/db/FASTA/nr.gz

6
http://www.phi-base.org/

7
http://www.tcdb.org/

8
http://geneontology.org/page/go-database

9
http://www.genome.jp/kegg/
